# A growing aneurysm of the posterior inferior cerebellar artery complicated with cerebellar infarction: A case report

**DOI:** 10.1016/j.ijscr.2021.106559

**Published:** 2021-11-02

**Authors:** Yuta Sasaki, Hiroki Yoshida, Hiroshi Horikawa, Keisuke Maruyama, Akio Noguchi, Yoshiaki Shiokawa

**Affiliations:** Department of Neurosurgery, Kyorin University Faculty of Medicine, Tokyo, Japan

**Keywords:** Arterial dissection, Cerebellar infraction, Diagnostic pitfall, Bypass surgery, Growing aneurysm, Etiology

## Abstract

**Introduction and importance:**

Hereby we describe an instructive patient with cerebellar infarction and a growing aneurysm at the posterior inferior cerebellar artery (PICA), which was not a true cause of infarction.

**Case presentation:**

A 50-year-old female presented with dizziness and posterior neck pain at our hospital (Mitaka city, Tokyo, Japan). Diffusion weighted magnetic resonance (MR) images showed cerebellar infarction in the left PICA territory and MR angiography study showed an aneurysm at the origin of the left PICA, which grew in 2 weeks. Since we considered cerebellar infarction was caused by thrombosis from the aneurysm, trapping of the PICA and occipital artery-PICA bypass was performed to prevent recurrent cerebellar infarction and rupture of the aneurysm by neurosurgeons. During the operation, dissection was observed at the distal PICA, which was diagnosed to be the true cause of cerebellar infarction. By the follow-up for 12 months at an outpatient, there was no recurrence of cerebral infarction.

**Clinical discussion:**

A specimen of the artery showing the findings of dissection was not obtained, and the pathological diagnosis could not be made. It would be controversial whether a surgical procedure presented here was the most optimal.

**Conclusion:**

This is a first reported case of growing aneurysms and cerebral infarction due to arterial dissection. Even if cerebral infarction is accompanied by growing aneurysms, arterial dissection should be included in the differential diagnoses of a cause of infarction. Posterior cervical pain can be a clue for early appropriate diagnosis in such a case.

## Highlights

• A patient with a growing aneurysm suffered from brain infarction with undiagnosed dissection.
• Even if an aneurysm grows, arterial dissection can be a true cause of infarction.
• Cervical pain can be a clue for diagnosing cause of infarction in such a case.

## 1 Introduction

The etiology of ischemic stroke is classified into the following five subtypes: 1) large-artery atherosclerosis, 2) cardio embolism, 3) small-vessel occlusion (lacunar), 4) stroke of other determined etiology, and 5) stroke of undetermined etiology [1].

Hereby the authors report an instructive patient with aneurysm at the posterior inferior cerebellar artery (PICA) complicated with cerebellar infarction whose true cause was finally turned out to be the dissection of the PICA directly observed at open surgery.

The incidence of arterial dissection that develops in the PICA alone was 0.5% to 0.7% of all intracranial aneurysms, and the most appropriate treatment has not yet been established [2]. While most of them presented with subarachnoid hemorrhage, and ischemic presentation was relatively rare [2]. Subarachnoid hemorrhage occurs when an aneurysm ruptures, and cerebral infarction occurs when a branching artery is occluded by a false lumen of dissection [3]. According to some studies of cerebral angiography of arterial dissection, there are few cases in which specific findings such as pearl and string signs and double lumen observed from the beginning of the onset [4], [5], [6].

A case of growing aneurysms and cerebral infarction due to arterial dissection has not been reported so far. Hereby the authors report an instructive patient with aneurysm at the posterior inferior cerebellar artery (PICA) complicated with cerebellar infarction whose true cause was finally turned out to be the dissection of the PICA directly observed at open surgery.

The work has been reported in line with the SCARE criteria [7].

## 2 Presentation of case

**Fig. 1:**
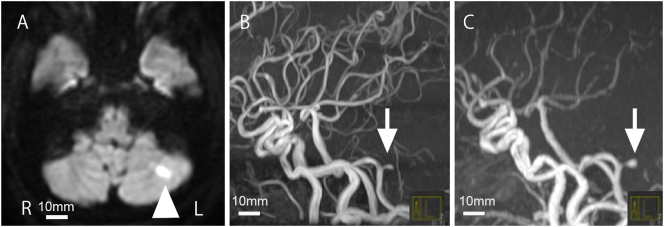
Preoperative brain MR images. A: A diffusion-weighted MR image showing acute cerebellar infarction in the left PICA territory (arrowhead). B–C: MR angiograms showing a saccular aneurysm at the origin of the PICA (B arrow), which increased in 2 weeks (C arrow).

A 50-year-old female with no past history, drug history, allergy, smoking, alcohol, and family history presented with acute dizziness and posterior neck pain. She visited an outpatient of another hospital (Chofu city, Tokyo, Japan) and had been diagnosed as cerebellar infarction at the left PICA territory by brain MR imaging (Fig. 1A). It showed a small aneurysm with a 2 mm diameter at the origin of the PICA (Fig. 1B) by MR angiography, which was found to be enlarged by a follow-up study 2 weeks later (Fig. 1C). The patient was referred to our institution (Mitaka city, Tokyo, Japan) for the treatment. Since source of embolism or arrhythmia was not evident in our search of stroke etiology, we considered that the most possible cause would be embolism due to thrombus from the aneurysm, and antiplatelet therapy was initiated. She was hospitalized for the treatment to prevent recurrence of cerebellar infarction and rupture of the aneurysm.

**Fig. 2:**
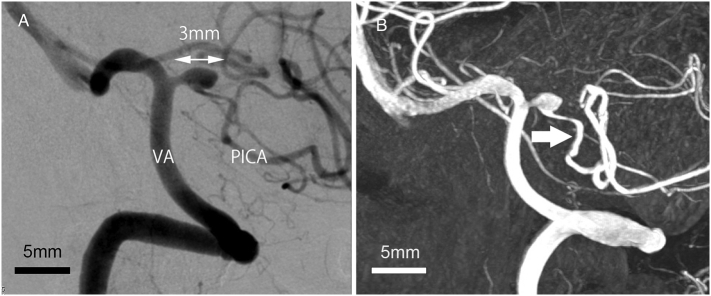
Left vertebral angiography, lateral view. A: A saccular aneurysm with a maximum diameter 3 mm at the origin of the left PICA.B: Distal PICA with vascular wall irregularities (arrow).

On admission, the patient was alert and showed dizziness but no cerebellar ataxia. Cerebral angiography showed vasodilation and pooling of contrast media just after the origin of the left PICA (Fig. 2). The vessel wall of the dilated PICA was irregular. Since the irregular vessel wall was considered to be within the range of arteriosclerosis, our preoperative diagnosis was a growing fusiform aneurysm at the PICA origin complicated with arteriosclerosis of the PICA. We did not lead to the diagnosis of dissection at this time.

**Fig. 3:**
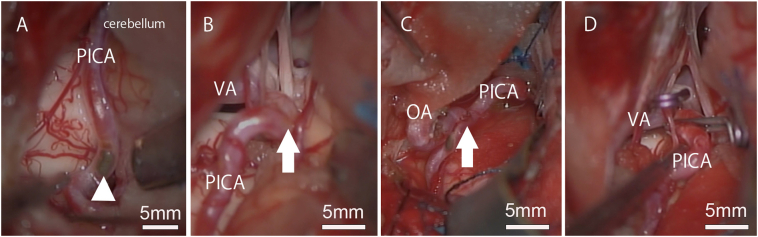
Intraoperative findings. A: Color change at distal PICA, suggestive of arterial dissection (arrowhead). B: A aneurysm at the origin of the PICA without any color irregularities suggestive of dissection (arrow). C, D: Bypass from the OA to the PICA (arrow indicates anastomosis) (C) and trapping the aneurysm (D).

After deliberate discussion on therapeutic options, occipital artery (OA)-PICA bypass and trapping of the aneurysm by lateral suboccipital approach was selected, since it seemed impossible to preserve sufficient blood flow of the distal PICA by standard aneurysm clipping. We also considered OA-PICA bypass can be a safe, effective, and highly customizable technique for the revascularization of patients with nonsaccular proximal PICA and VA-PICA aneurysms [8]. At surgery, irregularity and color change of the wall of the PICA proximal to the caudal loop was observed (Fig. 3A), which finally led to the diagnosis of dissection of the PICA. At the origin of the PICA, aneurysmal dilatation and localized color change was found (Fig. 3B). The aneurysm was diagnosed as dissection since morphologically it had atypical dilatation at the entire circumference and findings suspected of arterial dissection although it had no color change. Based on these findings, we considered the cause of cerebellar infarction would be dissection of the PICA. We performed OA-PICA bypass (Fig. 3C) and aneurysm trapping (Fig. 3D) as planned by a specialized neurosurgeon with 13-year experience. There was a small artery distal to the aneurysm running to the anterior-lateral direction, which was a possible branch to the brainstem. To preserve it, the part of the dissection had to be left as it was. We considered distal end of the dissection was the part of color change of vessel wall, because no color change was identified distal to it.

**Fig. 4:**
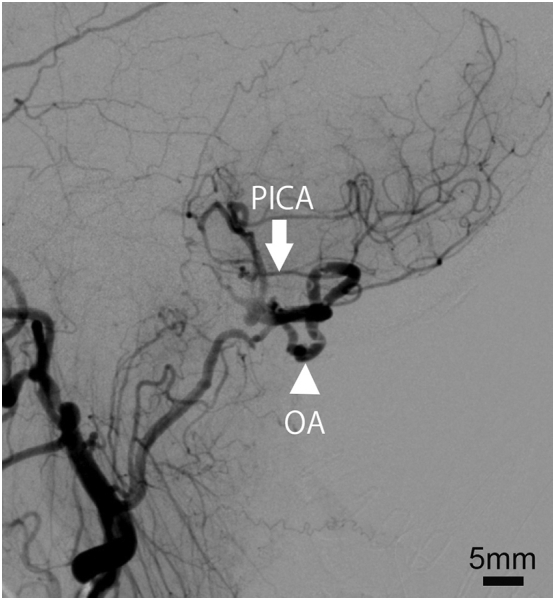
Postoperative left external carotid artery angiography, lateral view. The periphery of the PICA (arrow) was visualized through the anastomosed OA (arrowhead). No aneurysm was observed.

Postoperative angiography showed sufficient blood flow from the OA to the distal PICA and disappearance of the aneurysm (Fig. 4). No morphological change was found at the part of color change. Postoperative MR imaging showed no new infarction in the brain including the brainstem. Dizziness of the patient resolved and she was discharged 9 days after surgery while taking aspirin (A2093, Sigma-Aldrich, Meguro city, Tokyo, Japan) 100 mg per day for the prevention of recurrent ischemic stroke. No recurrence of stroke occurred for 12 months after surgery.

## 3 Discussion

We reported a patient with a growing PICA aneurysm and cerebellar infarction. Since a growing aneurysm was found at the PICA of the same side as cerebellar infarction, we first considered that the cerebellar infarction might be caused by the thrombus formation from the aneurysm. Distal embolism was reported to occur in 3.3% of intracranial aneurysms [9]. More than half of the aneurysms with distal embolism had a maximum diameter of 10 mm or less, of which the smallest one was 2 mm [9]. A mechanism of such distal embolism is that small aneurysms have a high blood flow, which increases the wall shear stress when forming a thrombus and inhibits the clot formation process, but may increase the risk of distal embolism [9]. We had considered the most probable cause of cerebral infarction in our patient might be thrombus formation in the aneurysm, and therefore, we performed a surgery for the purpose of recurrence of cerebral infarction. However, by observing under direct vision during the surgery, the findings of dissection such as irregular wall and color change of the distal PICA could be visually observed. As a result, it was diagnosed that this dissection was the true cause of cerebellar infarction. Whether dissection involved the aneurysm could not be confirmed because no pathological specimen was obtained.

The incidence of arterial dissection that develops in the PICA alone was 0.5% to 0.7% of all intracranial aneurysms, and the most appropriate treatment has not yet been established [2]. While most of them presented with subarachnoid hemorrhage, and ischemic presentation was relatively rare [2]. Also most frequent symptoms were headache, which was often difficult to distinguish from primary headache [10]. Subarachnoid hemorrhage occurs when an aneurysm ruptures, and cerebral infarction occurs when a branching artery is occluded by a false lumen of dissection [3]. It might be often difficult to diagnose when headache is the only symptom at onset, and diagnosis has to be made based on the current medical history and imaging findings without a definitive diagnosis.

At the time of preoperative angiography, we only recognized irregularity of the wall of the PICA and could not lead to the diagnosis of dissection. Diagnostic criteria of cerebral dissecting aneurysms are as either of the following findings; (1) double lumen and pearl and string sign confirmed by cerebral angiography, (2) proof of intramural hematoma by MR imaging, (3) publish red or dark red appearance of the aneurysm, or (4) pathological proof [11]. In our patient, the above (1) and (2) were not met before surgery, and the diagnosis was finally reached only by (3). Since the incidence of arterial dissection involving the PICA alone is relatively low as described above, it had not been easy to suspect dissection before surgery. On the other hand, according to some studies of cerebral angiography of arterial dissection, there are few cases in which specific findings such as pearl and string signs and double lumen observed from the beginning of the onset [4], [5], [6]. Instead, only non-specific findings such as irregular walls, stenosis, or occlusion were observed, and typical findings appeared later [6]. Therefore, if irregularity, stenosis, or obstruction of the arterial wall was observed by cerebral angiography, it would be necessary to keep in mind that they may be signs of dissection [6]. In addition, posterior neck pain, seen in our patient at the first visit, could be another clue for early diagnosis since it was observed in 42% of patients with arterial dissection at posterior circulation [2].

There has been no report of patients with growing aneurysms and cerebral infarction due to arterial dissection in our knowledge. We reported here since this report could be didactic to illustrate diagnostic pitfall. It is well-known that arterial dissection can be the cause of ischemic stroke, and the incidence of vertebral artery dissection was conventionally 2 to 3 out of 100,000 population, which has increased with the progress of diagnostic imaging in recent years [2].

This report has some limitations. First, a specimen of the artery showing the findings of dissection was not obtained, and the pathological diagnosis could not be made. However, it was not realistic to resect all or part of the pathological area of the artery to maintain blood flow through the bypass. Second, it would be controversial whether a surgical procedure presented here was the most optimal. Bypass surgery was planned first, and the procedure was performed as planned after the eventual diagnosis of dissection during the operation. To prevent recurrent cerebral infarction, more ideal option would be complete disruption of blood flow at the part of arterial dissection and bypass at the site without dissection, which was hard to perform in our patient.

## 4 Conclusion

A case of growing aneurysms and cerebral infarction due to arterial dissection has not been reported so far. Even if cerebral infarction is accompanied by growing aneurysms, arterial dissection should be included in the differential diagnoses of a cause of infarction. Posterior cervical pain can be a clue for early appropriate diagnosis in such a case.

## Guarantor

Yuta Sasaki.

## Sources of fundings

This research did not receive any specific grant from funding agencies in the public, commercial, or not-for-profit sectors.

## Ethical approval

This report describes established surgical procedures and therefore does not require ethical approval at our institution (Kyorin University).

## Consent

Written informed consent was obtained from the patient for publication of this case report and accompanying images. A copy of the written consent is available for review by the Editor-in-Chief of this journal on request.

## Provenance and peer review

Not commissioned, externally peer-reviewed.

## Research registration

Not applicable.

## CRediT authorship contribution statement

Yuta Sasaki: writing-preparation of the original draft, operating surgeon.

Hiroki Yoshida: operating surgeon.

Hiroshi Horikawa: supervision.

Keisuke Maruyama: critically revising the manuscript.

Akio Noguchi: supervision.

Yoshiaki Shiokawa: supervision.

## Declaration of competing interest

No.
